# Development of a symptom menu to facilitate Goal Attainment Scaling in adults with Down syndrome-associated Alzheimer’s disease: a qualitative study to identify meaningful symptoms

**DOI:** 10.1186/s41687-020-00278-7

**Published:** 2021-01-11

**Authors:** Kari Knox, Justin Stanley, James A. Hendrix, Hampus Hillerstrom, Taylor Dunn, Jillian Achenbach, Brian A. Chicoine, Florence Lai, Ira Lott, Sanja Stanojevic, Susan E. Howlett, Kenneth Rockwood

**Affiliations:** 1 DGI Clinical Inc, 300SH-1701 Hollis St, Halifax, NS B3J 3M8 Canada; 2 LuMind IDSC Foundation, Burlington, MA USA; 3 Dalhousie University, Halifax, NS Canada; 4 Advocate Medical Group, Park Ridge, IL USA; 5 Massachusetts General Hospital, Harvard University, Boston, MA USA; 6 University of California Irvine Institute for Memory Impairments and Neurological Disorders, Irvine, CA USA

**Keywords:** Down syndrome, Alzheimer’s disease, Dementia, Goal attainment scaling, Symptoms, Qualitative study

## Abstract

**Background:**

As life expectancy of people with Down syndrome (DS) increases, so does the risk of Alzheimer’s disease (AD). Identifying symptoms and tracking disease progression is especially challenging whenever levels of function vary before the onset of dementia. Goal Attainment Scaling (GAS), an individualized patient-reported outcome, can aid in monitoring disease progression and treatment effectiveness in adults with DS. Here, with clinical input, a validated dementia symptom menu was revised to facilitate GAS in adults living with Down Syndrome-associated Alzheimer’s disease (DS-AD).

**Methods:**

Four clinicians with expertise in DS-AD and ten caregivers of adults living with DS-AD participated in semi-structured interviews to review the menu. Each participant reviewed 9–15 goal areas to assess their clarity and comprehensiveness. Responses were systematically and independently coded by two researchers as ‘clear’, ‘modify’, ‘remove’ or ‘new’. Caregivers were encouraged to suggest additional items and recommend changes to clarify items.

**Results:**

Median caregiver age was 65 years (range 54–77). Most were female (9/10) with ≥15 years of education (10/10). Adults with DS-AD had a median age of 58 years (range 52–61) and either a formal diagnosis (6/10) or clinical suspicion (4/10) of dementia. The initial symptom menu consisted of 67 symptoms each with 2–12 descriptors (589 total). The clinicians’ adaptation yielded 58 symptoms each with 4–17 descriptors (580 total). Of these 580 descriptors, caregivers identified 37 (6%) as unclear; these were reworded, and one goal area (4 descriptors) was removed. A further 47 descriptors and one goal area were added to include caregiver-identified concepts. The final menu contained 58 goal areas, each with 7–17 descriptors (623 total).

**Conclusions:**

A comprehensive symptom menu for adults living with DS-AD was developed to facilitate GAS. Incorporating expert clinician opinion and input from caregivers of adults with DS-AD identified meaningful items that incorporate patient/caregiver perspectives.

## Plain English summary

The prevalence of Alzheimer’s disease is above 75% in people with Down syndrome aged 60 years or older. There is a need for novel methods to evaluate disease presentation and progression that are applicable to people with intellectual or developmental disabilities. Identifying and tracking dementia symptoms is difficult and even more so in people with Down syndrome.

In this study, we developed a dementia symptom menu specific to people with Down syndrome to make symptom tracking easier in this population. We interviewed caregivers and doctors who have expertise in Down syndrome-associated Alzheimer’s disease to develop a comprehensive symptom menu specific to people with Down syndrome and dementia. The menu consisted of 58 symptoms related to different areas such as cognitive decline (e.g. recent memory), behavioural changes (e.g. irritability/frustration), and physical changes (e.g. incontinence). Each symptom also included about a dozen plain language descriptions of possible symptom presentations.

By including both clinical opinion and input from caregivers of adults with Down syndrome-associated Alzheimer’s disease, we were able to identify a list of meaningful symptoms that include patient and caregiver viewpoints.

## Background

People with Down syndrome (DS) are living well into their 50s and 60s with the median life expectancy having risen to 58 years in the US [1]. This increased life expectancy comes with an increased, age-related risk of developing Alzheimer’s disease (AD) [2]. Current estimates place the prevalence of AD above 75% in people with DS aged 60 years or older [3–6]. Evaluating health outcomes in people with DS is already difficult due to varying levels of baseline function and cognition [7]. This complexity is further exacerbated by the onset of dementia [8]. For example, managing finances and driving are commonly assessed activities of daily living in dementia but a person with DS might not ever have been able to perform such a task [7]. It is therefore important to consider baseline performance in these individuals [7, 8]. There is also growing evidence that adults with Down syndrome-associated Alzheimer’s disease (DS-AD) respond differently to treatment when compared to people with dementia who do not have DS [9]. In consequence, there is a need for measures that capture heterogeneous disease presentation and treatment response in those with DS and dementia [10–13]. For this, individualized patient-reported outcomes are well-suited [14, 15].

Goal Attainment Scaling (GAS) is an individualized outcome measure initially established for mental health disorders [16]. GAS has been applied to various conditions and disease states including dementia [15–19]. It enables patients and their caregivers to identify and track goals of treatment that are meaningful to them and are related to their condition. GAS is feasible in a variety of applications [20–31], even though the goal-setting process is sometimes described as difficult and time-consuming [32–35]. It can sometimes be difficult to be both patient-centred, and to make explicit clinical judgments about the attainability of goals. These issues are often addressed through training programs designed for clinicians administering GAS [34, 36]. A complementary approach develops condition-specific goal menus to facilitate goal setting by patients, their caregivers and/or clinicians [30, 37, 38].

One such menu is available in SymptomGuide®-dementia, an online platform for people living with dementia and (more often) their care partners to track changes in the symptoms that are most important to them [19]. The menu of 67 dementia symptoms was developed through patient, clinician and caregiver feedback, and the data obtained from over 4000 SymptomGuide® users. It has been used to gain insights into common dementia symptoms such as verbal repetition [39] and agitation [40], and, as with GAS, has been found to be sensitive to change [41, 42]. This menu, however, is not specifically tailored towards people with DS. In this study, our aims were to adapt the SymptomGuide®-dementia menu to facilitate Goal Attainment Scaling in adults with DS-AD and to identify challenges most meaningful to people with DS-AD and their caregivers.

## Methods

### Dementia symptom library

The SymptomGuide®-dementia contains information on 67 dementia symptoms that consists of 2–12 (589 total) specific descriptions of their possible manifestations, called descriptors. Alternatively, users can define their own unique symptoms or descriptors not included in the menu. Variations of this menu have been used in a clinical study for people with vascular or mixed AD/vascular dementia [41] and in a tertiary care memory clinic [43] to track disease progression. In this study, we elicited feedback from caregivers of adults living with DS-AD and clinicians who have expertise in DS to tailor the SymptomGuide®-dementia symptom library for use in this population.

### Sample

The expert panel consisted of four clinicians (JA, BAC, FL, IL) with expertise in DS-AD. Two researchers (KK, a research nurse and KR, a physician) conducted semi-structured qualitative interviews with them. To ensure the menu’s clarity and comprehensiveness, 10 people were recruited who cared for someone with both DS and a formal diagnosis or clinical suspicion of dementia to participate in individual semi-structured interviews. Semi-structured qualitative interviews with caregivers were conducted by KK.

For this qualitative study, purposive sampling was used to recruit people most likely to use this menu in practice: English speaking caregivers (typical case sampling) and clinicians (expert sampling). Participants were recruited from the United States or Canada. The sample size followed guidelines of the International Society for Pharmacoeconomics and Outcomes Research Patient-Reported Outcomes Good Research Practices Task Force [44, 45]. Caregivers most often reported caring for women with DS-AD, which is consistent with gender effects on AD in adults with DS [46]. The interviews were conducted between June and December 2019.

All interviews were audio-recorded and transcribed verbatim. Grounded Theory, an approach that applies inductive reasoning to systematically acquired data [47], was used to conceptualize the symptoms most meaningful to adults living with DS-AD and their caregivers. Grounded theory was used to identify potential symptoms described spontaneously and during menu review to revise the SymptomGuide®-dementia menu for this population. Our aim in developing individualized measures to assay disease progression and treatment effects was to achieve an adjudication that was relevant and non-arbitrary at the individual patient level.

### Clinician interviews

The first clinician interview was conducted individually, in-person; the second was conducted in an online group setting with the remaining three clinicians. These interviews were used to determine whether SymptomGuide®-dementia was comprehensive and reflected the DS population. Clinicians were asked the following: 1) to describe the most important dementia symptoms identified by adults living with DS-AD or their carers; 2) to identify new symptoms and descriptors that were not available in SymptomGuide®-dementia; and 3) to ensure that the symptoms and descriptors were written in language that was easy to understand and to use. All suggested menu revisions were discussed in subsequent caregiver interviews. The interview facilitators reviewed audio recordings and interview transcripts to ensure all clinician-identified themes were present in the revised menu.

### Caregiver interviews

Caregivers were recommended by the LuMind IDSC Foundation or recruited through social media. Potential participants were screened based on inclusion/exclusion criteria (Appendix). Each semi-structured caregiver interview was divided into four sections (Table 1). Interviews were conducted via online video conferencing to facilitate slide sharing and audio recording. Each interview lasted approximately 1.5 h. To ensure rigour, the nurse used an interview guide, took notes, and actively solicited participant feedback during interviews. The research nurse’s reflections were documented following each interview to remove biases when responding to participants and to improve the interviewing style for subsequent interviews.

**Table 1:** Caregiver interview structure

Section	Structure	Description	Time (mins)
1	Structured	Introduction to the interview and review of consent form. Participants provided verbal consent and demographic data.	15
2	Open-ended	Discussion of the day-to-day symptoms and challenges faced when caring for someone with DS-AD.	30
3	Structured	In-depth review and feedback on a subset of symptoms including each of their descriptors. Participants were given the opportunity to provide additional items and suggest changes to unclear items.	30
4	Open-ended	Discussion of the most important symptoms and challenges to the individual participant with respect to DS-AD.	15

### Menu revision

Both clinicians and caregivers were asked to discuss the most common and meaningful challenges they experienced while caring for someone with DS-AD. They were then prompted to provide both examples of symptoms from dementia onset as well as their current symptoms.

During the interviews, caregivers were shown a randomly selected subset of 9–15 SymptomGuide®-dementia symptoms and their descriptors. Each item was read aloud one-by-one, pausing for caregiver responses between items. Caregivers were asked to report whether the items were clear and in language that they would typically use. They were given the opportunity to suggest alternative wording for menu items and, after reviewing each symptom, they were offered the chance to identify concepts not included in the menu. Each menu item was reviewed by 2–3 caregivers. Following symptom review, caregivers were shown the full menu to assess the clarity, comprehensiveness, and comprehensibility of the symptom list. Caregivers were then asked to identify the five symptoms most important to them. They were advised that they were not bound by the symptom menu and could identify their own unique symptoms.

### Data analysis

Concepts from the clinician interviews were reviewed and discussed by both the research nurse and physician facilitator until a consensus was reached. Identified concepts were compared against existing menu items. New symptoms, descriptors and revisions were implemented prior to caregiver interviews. Two researchers listened to audio recordings of caregiver interviews while reading through interview transcripts to familiarize themselves with the data. Caregiver feedback on menu items were extracted, arranged by symptom and descriptor and then coded as ‘clear’, ‘modify’, ‘remove’ or ‘new’. Researchers discussed codes for each menu item until a consensus was reached. Considering the individualized nature of the menu (for which each item need not be relevant to every individual), some items were added or modified based on a minority of participant comments. To ensure the trustworthiness of the results, a rigorous approach that included reflexivity (the researcher reflects on the processes used for data collection and interpretation) and an audit trail (complete description of research steps taken) for all data analyses was used. Opinions were solicited from a range of stakeholders (e.g. clinicians, caregivers, study authors, etc.) and included open-ended discussion to reduce bias.

Quotes from the open-ended portions of caregiver interviews were independently analyzed by two researchers using NVivo 12 qualitative data analysis software (QSR International Pty Ltd.). Data were categorized into key themes using a seven-step process: transcription, data familiarization, coding, identification of a thematic framework, indexing, mapping, and interpretation [48]. Two researchers independently reviewed audio-recordings and interview transcripts. Researchers then independently indexed quotes not directly related to menu review. Quotes were then discussed to develop an initial thematic framework and coding structure using both inductive and deductive approaches. Quotes were then coded under each theme. Researchers compared codes and discussed any new findings. Codes were mapped and interpreted to determine the key findings.

Demographic data were extracted from deidentified interview transcripts and summarized by means and standard deviations, medians, and ranges, or by frequency and proportions as appropriate.

### Ethics

This study was approved by WIRB Copernicus Group Inc. research ethics board (study #1265203). All participants provided informed consent.

## Results

### Subject characteristics

The caregivers of adults with DS (*n*=10) ranged from 54 to 77 years (median 65 years). All were siblings of the person with DS-AD that they cared for and most were women (9/10) with ≥15 years of education (10/10). The 10 adults with DS-AD were between 52 and 61 years of age (median 58 years) with a formal diagnosis (6/10) or clinical suspicion (4/10) of dementia. The people with DS-AD lived either with the caregiver interviewed (7/10) or in a group home (3/10). The demographic information is summarized in Table 2.

**Table 2:** Characteristics of the person with DS and their caregiver

Characteristic	Participants (***N***=10)
Caregiver	
Age, median (range) in years	65 (54–77)
Gender, % (N) women	90% (9)
Education, % (N) ≥15 years	100% (10)
Adult with DS-AD	
Age, median (range) in years	58 (52–61)
Gender, % (N) women	70% (7)
Education, % (N) ≥12 years	70% (7)
Relationship to caregiver, % (N) Sibling	100% (10)
Dementia, % (N) clinically diagnosed	60% (6)

### Menu revisions

Expert clinician interviews resulted in several revisions. Of the original 67 SymptomGuide®-dementia symptoms, 5 (7%) were deemed ‘not relevant’ to people with DS and were removed (e.g. Looking after grandchildren). Some, (*n*=5, 7%) were renamed to better reflect difficulties associated with caring for someone with DS (e.g. ‘Travel’ renamed to ‘Travel and Transportation’). The content of 12 symptoms (18%) was combined resulting in 6 symptoms (e.g. ‘Appetite’ and ‘Eating’ were combined into ‘Eating’). In addition, ‘Money/Math’ was added to the menu in lieu of ‘Financial Management’. ‘Vocational (work)’ was also added to describe decline in gainful employment performance or group home tasks. With respect to the original 589 descriptors, 24 (4.1%) were reworded and 102 (17.3%) were removed. A further 93 descriptors were added. This resulted in a revised menu containing 58 symptoms each with 4–17 descriptors (580 total) tailored to individuals with DS-AD.

Caregivers reviewed a median of 11.5 symptoms (range 9–15) on the revised menu. Each symptom was reviewed by 2–3 caregivers (median 2). Most symptoms 98% (57/58) were endorsed by the caregivers. The “Money/Math” symptom was not endorsed and was therefore removed from the menu. Caregivers commented that the adults with DS they cared for only had a basic understanding of money and math even before the onset of dementia.

“ … if you just said, ‘this costs two dollars and ten cents’ she wouldn't know how to do that and she would never know if somebody gave her the correct change or not.”

“ … she would get her own money out for certain things just because it was habit. She had learned that, when it was bowling day, she needed three one-dollar bills and two quarters.”

“When she first started working in the shelter workshop, she was going to get a paycheck. Her big thing was she was taking everybody out for McDonald's hamburger, French fries, Coke, everybody can have one. And so, you know, she had a dollar sixty-eight.” The caregiver then sarcastically remarked “Six brothers and sisters and a mother and father and we all went out for dinner on a dollar sixty-eight … Not.”

Descriptors from this goal that were deemed relevant were moved to other goals, for example, “Trouble paying with debit” was moved to the “Shopping” symptom. Additionally, a symptom for “Seizures” was added to the menu. Most caregivers (6/10) reported seizures and described seizure-like episodes as traumatic events for both themselves and the person they cared for.

“He has had some incidents and I don't know what it is... He'll call out and say, “Come here, come here, come here”. I get there, he passes out cold on the floor, his eyes roll up in his head.”

“The seizures are a pretty dramatic event. And for people that are not in the medical profession, seizures are downright scary.”

A further six symptoms were renamed based on caregiver feedback. For example, ‘Operating gadgets/appliances’ was renamed to ‘Operating devices/appliances’ to reflect current nomenclature and ‘Meal preparation and baking’ was renamed to ‘Meal-time preparation and activities’ to reflect the level of function in adults with DS.

Most of the descriptors in the revised menu (93%, 539/580) were endorsed by caregivers. After reviewing the ‘Irritability/frustration’ symptom, one caregiver said, “It’s like they’re written for her!”. While reviewing the clarity of descriptors in the ‘Eating’ symptom, another commented “Oh my God, [the descriptor is] clear, I don’t feel like I’m crazy anymore” after being shown the descriptor ‘Gags and clears throat frequently’. The individualized nature of this tool allows for the inclusion of less common symptom manifestations. One caregiver supported this method by spontaneously saying,

“So, you know, some of these things I'm surprised to see them here in writing during a research study … I didn't realize it was common enough to be on the questionnaire”

Descriptors that were unclear to caregivers (*n*=37, 6%) were reworded; those that were not endorsed were removed (*n*=4, 1%). A further 46 descriptors were added to include caregiver-identified concepts. Examples of additional menu revisions can be found in Table 3. The final menu contained 58 symptoms, each with 7–17 descriptors (622 total).

**Table 3:** Examples of menu revisions based on caregiver feedback

Symptom	Descriptor	Caregiver Feedback	Modified/New Descriptor
Memory for Names and Faces	Cannot name well known public figures	“This was true of my sister and all the people I know with Down syndrome. They have celebrities who they adore. ...And that became an issue, like recognizing some of her favorite [baseball] players and losing interest in them.”	Cannot name well known public figures or celebrities
Personality Changes	Becomes sad	“I know depression is a problem. Which is pretty much. Well it’s not really the same thing but people might recognize it as the same thing.”	Becomes sad or depressed
Eating	Only eats with a spoon	“Or doesn’t use a utensil. That’s our new thing. She’d rather eat with her fingers. So, we went from silverware to … to nothing now.”	Unable to use utensils, therefore uses hands/fingers to eat
Self-Awareness and Insight	Unable to describe their feelings	“Maybe unable to “name” or describe their feelings? Because sometimes it’s a word retrieval thing. I can’t think of the word angry, but I can tell you that I feel like hitting you.”	Unable to name or describe their feelings
Dressing	–	“He insists on wearing the same pair of shorts all week. And if he’s dressed and has a spot on his shorts and I say, “You can’t wear those today. Look, they’re dirty.” He goes “they’re not dirty, they’re not dirty” and it’s a whole issue.”	Wants to wear favourite outfit everyday

### Thematic analysis

Chiefly, two key findings emerged from the thematic framework analysis: caregiver burden and healthcare difficulties. Caregiver burden was expressed in many ways such as increased responsibilities and financial impacts:

“I just realized that it was just too much … And so, I left my job in 2016 and I became her full-time caregiver.”

“There was no program here in our community that I could send her to. So, then I had to start paying people to stay with her during the day.”

“About a year after she was there … she started to display a lot of things and I was out of sight of my family. So, we relocated back to our original state. And that sort of improved things for a while but certain symptoms and challenges continued but at least I have more family to assist me.”

“She goes to the mall, she goes to the movies, she goes to the library and we try to go out at least to a senior center. She'd rather stay in the house, but she has to go out. It's a lot of my time but she has to get out, so I just make it happen.”

Some caregivers confided the emotional hardships they experienced when caring for someone with DS-AD.

“It's gut wrenching for me, to be honest. I mean she was always just the sweetest person and never would have hit somebody in a million years. And now she wants to hit people; she wants to hit me too.”

“I was scared to death; I could see what was happening and I just it was like “my God” … this is bad but it's going to be so much worse.”

In the second theme, caregivers described challenges with medical professionals who were not well-equipped for patients with DS-AD.

“I talk with [doctor] every time we go, and we discuss the changes that I’m seeing, the regression that I’m seeing … I don’t know that she knows a whole lot about people with Down syndrome. I asked her, ‘Do you have any other patients with Down syndrome?’ and I think she said she’s had two other ones.”

“I was shocked at what he couldn’t do but I also was not impressed with the person who gave it [test] to him. We walk out and he says, “so tell me, he does have Down syndrome, right?” I'm like “What the heck are you asking me? Take a look. You got to ask me?”

This was particularly noted by caregivers living in smaller communities.

“I think that was more of a result of where we live. You know small rural town and, when I went to nearby towns, larger towns that had nice medical centers, we've been able to get exactly what we need. It was just the travel that was difficult.

**Fig. 1:**
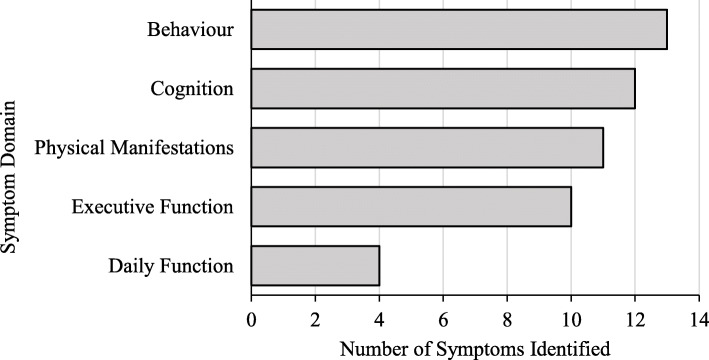
Symptom domains identified as most important. The number of symptoms reported was plotted as a function of the specific symptom domain categories caregivers identified as most important in this study. Symptoms related to daily function were least often regarded as important by the caregivers

To close interviews, caregivers were asked to identify five symptoms that were most important to them. They were shown a list of all symptoms included in the revised menu however, they were advised that the symptoms they identify as most important need not be selected from the menu. A total of 25 unique important symptoms were identified, all of which had been included in the menu. The most common were ‘Anxiety and Worry’, ‘General Memory’ and ‘Incontinence’ each reported by 4/10 participants. We categorized caregiver-reported symptoms into five domains: behaviour, cognition, daily function, executive function, and physical manifestations. Caregivers most often identified changes in behaviour (13/50, e.g. irritability/frustration) followed by cognitive decline (12/50, e.g. verbal repetition) and physical manifestations (11/50, e.g. incontinence; Fig. 1).

## Discussion

The aim of this study was to adapt the SymptomGuide®-dementia symptom menu for use in adults living with DS-AD. Expert clinicians and caregivers of people affected by DS-AD were interviewed to ensure that modifications were meaningful to this population. First, menu modifications were made after eliciting feedback from expert clinicians. Their expertise guided the revision or addition of over 200 menu items. Feedback from a representative sample of caregivers was then elicited which resulted in nearly 100 additional changes. This resulted in a comprehensive symptom menu that was specifically tailored for adults with DS-AD and their caregivers. This work shows that input from both expert clinicians and caregivers can be used to identify symptoms that are most meaningful both to people living with DS-AD and to their caregivers. The resulting menu will be used to facilitate the use of GAS in adults with DS-AD.

Interestingly, some items revised based on clinician feedback were further modified or even changed back to the original form based on caregiver feedback. For example, the symptom titled ‘Meal Preparation and Cooking’ was first changed to ‘Meal Preparation and Baking’ based on clinician feedback and later changed to ‘Meal-Time Preparation and Activities’ based on caregiver feedback. This highlights the need for real world evidence and patient engagement in the early stages of patient-centred outcome development. Indeed, the US Food and Drug Administration recommends early patient involvement in new Patient-Reported Outcome measures [49]. By asking stakeholders to describe both early and present symptom manifestations in this study, we were able to develop a comprehensive menu applicable to any level of impairment. The symptoms that each individual targets however will not only vary by personal preference but also by dementia stage. For example, problems with recent memory most often appear in early stages of dementia where symptoms are mild, whereas hallucinations do not usually emerge until the disease progresses into later stages [50–52]. Moreover, the nature of each symptom likely also captures disease progression. For example, in the symptom ‘misplacing objects’ the descriptor most used at the early stage commonly reflects simply mislaying items, compared with the later stages, in which odd placement of items (e.g. placing eye-glasses in the freezer) is more likely [53]. Each item on the menu need not be meaningful to every individual. Even so, the menu is designed to be robust enough such that everyone can find personally meaningful symptoms and descriptions no matter the level of impairment. In this study we did not consider dementia stage, but this could be interesting to explore in future work.

The baseline level of cognition, function, and behaviour in people with DS varies greatly within the DS population and even more so between adults with DS-AD and other people with dementia [7, 8, 10–13]. When asked to report the most meaningful challenges faced when caring for someone with DS-AD, caregivers chiefly described an enhanced sense of burden in addition to the challenges associated with disease progression. Caregivers described many different causes for their sense of burden, again demonstrating important heterogeneity in this population. Many caregivers reported needing to leave the workforce. Consistent with this, caregivers can average 52 h per week caring for someone with both dementia and intellectual or developmental disabilities [54]. Together these observations underscore the challenges posed by caring for adults who live with DS-AD.

Although cognitive decline is the hallmark of dementia in people without DS, as in other informant-based studies [55], caregivers in this study frequently reported changes in behaviour and physical manifestations as its most meaningful impacts. This can pose problems when applying outcome measures designed to assess the decline in late-life cognitive function to assess intervention or track the disease course in adults living with DS-AD. Much of the difficulty from the marked heterogeneity in cognition, function, and behaviour of this group occurs prior to the onset of dementia. In addition, standardized measures that focus on changes in cognition or daily function might not capture the changes that are most meaningful to the people with DS-AD or their caregivers. In consequence, individualized measures for evaluating disease progression and symptomatic changes that are specific to adults with DS-AD are needed. The symptom menu developed in this study provides an opportunity to increase the uptake and use of one such measure, GAS. Comparable to measures of global change, GAS provides an overarching metric of change. The standardized GAS T-score evaluates the extent to which goals have been met [16] – or in this case, the extent to which symptoms have improved. However, unlike measures of global change that do not specify the source of what has changed (e.g. the Clinician Interview Based Impression of Change [56]), tracking symptom-defined goals with GAS can provide an opportunity to determine specifically which symptoms are influenced by an intervention. That is, participants would select a set of symptoms and track changes in those symptoms individually over regular intervals depending on the study or intervention. For example, subjects might track changes once every 3 months. Moreover, another strength of GAS is that the methodology can be equally applied to individuals with vastly different backgrounds. GAS scales are uniquely developed for each individual and can therefore readily accommodate cultural differences between generations, geographic locations, and socioeconomic status.

Although simplified and menu-facilitated GAS has been shown to be feasible in several conditions [30, 37, 38, 41, 57], further work is needed to assess the responsiveness, feasibility and validity of the DS-AD menu in this population. To do so, the next step in this process is to evaluate GAS facilitated by this menu in a feasibility study or as an exploratory outcome measure in adults living with DS-AD. Furthermore, GAS is designed for individualization such that goal attainment is rated based on participant-defined concepts. Other important symptoms may become apparent if other methods of data collection are used such as focus groups, face-to-face interviews, or clinic visits. Given data from other studies, we will have the opportunity to describe and include newly identified concepts in the menu. In addition to its use in clinical studies, it is also important to note other potential applications for the menu that can be conducive to clinical care. SymptomGuide®-dementia is currently used in a memory clinic as a shared decision-making tool to help clinicians identify and track change in the symptoms most troubling to their patients [43]. This digital symptom tracking tool can be even more helpful for patients and caregivers who live in rural or remote areas and are therefore unable to see their clinicians regularly. Symptom data collected in this way could also be used in conjunction with other clinical assessments. For example, relationships between patterns of dementia symptom tracking and stage have been identified using an artificial neural network [58]. The use of this menu could likewise help better understand dementia progression in adults living with DS-AD.

## Conclusions

In conclusion, this study demonstrated that a comprehensive dementia symptom menu could be developed specifically for adults living with DS-AD. The menu was developed with expert clinician input and with feedback from caregivers of those with DS-AD. Input from these two groups allowed us to identify meaningful items that incorporate the perspectives of the people with DS and their caregivers. This menu will ultimately help facilitate the use of GAS, an individualized patient-reported outcome measure, to monitor dementia progression and the effectiveness of treatment in this population.

## Data Availability

De-identified participant data including supporting quotes will be made available to researchers upon reasonable request. Requesters who wish to access data must enter a data access agreement with the LuMind IDSC Foundation.

## Appendix

### Criteria for Participant Selection

**Recruitment Targets:**

- Population: Caregivers of people with both Down syndrome and a diagnosis or clinical suspicion of dementia
- Geographic location: United States of America
- Age: Adult caregivers aged 18 years or older; Person with Down syndrome and dementia aged 45 years or older
- Gender: Men and Women
- Race: Any
- Number of participants: 8–10
- Selection method: Purposive sampling

**Inclusion Criteria:**

***Caregiver:***

- Aged 18 years or older
- English speaking
- Access to computer with internet access
- Currently cares for or had recently cared for someone with both Down syndrome and dementia
- Willing and able to discuss symptoms, changes, and challenges with respect to Down syndrome and dementia.
- Familiar with the person with Down syndrome prior to dementia onset

***Person with Down syndrome and dementia:***

- Aged 45 years or older
- Diagnosed with Down syndrome
- Diagnosed with or clinical suspicion of dementia

**Exclusion Criteria**

***Caregiver:***

- Inability to complete interview in English
- Unable to meet technical requirements (e.g. unable to connect microphone, speakers, or telephone with video conferencing tool)
- Inability/refusal to meet scheduling needs of interviewer
- Refusal to provide informed consent

## Abbreviations

AD: Alzheimer’s disease
DS: Down syndrome
DS-AD: Down syndrome-associated Alzheimer’s disease
GAS: Goal Attainment Scaling
US: United States
